# Channel-resolved excitation trends in Si, GaAs, and NiO for sub-GeV dark matter detection

**DOI:** 10.1038/s41598-026-61355-3

**Published:** 2026-07-16

**Authors:** M. A. M. Sharaf

**Affiliations:** Department of Mathematics, Faculty of Science, Ain Shams University, Cairo, 11566 Egypt

**Keywords:** Sub-GeV dark matter, Light dark matter detection, Material-dependent detection, Multi-channel response, Electronic excitations, Phonon excitations, Magnon excitations, Condensed matter detectors, Silicon, Gallium arsenide, Nickel oxide, Materials science, Physics

## Abstract

The direct detection of sub-GeV dark matter requires target systems capable of converting very small deposited energies into observable low-energy excitations. In this work, we develop a phenomenological comparative framework for examining electronic, phononic, and magnonic response trends in three representative condensed-matter targets: silicon (Si), gallium arsenide (GaAs), and nickel oxide (NiO). The purpose of the framework is not to provide an ab initio prediction of absolute event rates, but to compare how material-specific excitation scales and effective channel weights influence the relative response under a common set of dark matter assumptions. The total response is decomposed into channel-resolved contributions and expressed in terms of normalized response rates and fractional channel measures. To address the model dependence of the effective channel weights, we supplement the baseline calculation with a weight-sensitivity analysis in which the leading channel is penalized while competing channels are enhanced. Within the adopted literature-guided phenomenological parameterization, Si remains electron-dominated, GaAs remains phonon-dominated under moderate adverse perturbations, and NiO remains magnon-dominated. However, the unit-weight test shows that the GaAs classification is conditional on including material-guided channel weighting, emphasizing that the present results should be interpreted as normalized comparative trends rather than absolute detector-rate predictions. The analysis therefore supports a cautious material-specialization picture: semiconducting, polar, and magnetic targets can preferentially emphasize different low-energy excitation signatures, but the quantitative hierarchy depends on the adopted response functions, channel weights, thresholds, and interaction assumptions. The study provides a transparent baseline for organizing multi-target detection strategies and identifies the main limitations that must be addressed by future microscopic or ab initio calculations.

## Introduction

The nature of dark matter remains one of the most fundamental open problems in modern physics. While the weakly interacting massive particle (WIMP) paradigm has historically guided experimental efforts, increasing attention has shifted in recent years toward sub-GeV dark matter candidates. In this low-mass regime, the energy transferred in scattering events is typically too small to be detected using conventional nuclear recoil techniques, motivating the exploration of alternative detection strategies based on low-energy excitations in condensed-matter systems, where the relevant energy scales naturally match those of sub-GeV dark matter interactions^1,2^.

Condensed-matter targets provide a versatile platform for probing light dark matter through a variety of excitation channels. Depending on the microscopic structure of the material, dark matter interactions may produce electronic excitations, lattice vibrations, or collective spin excitations. These mechanisms have led to several complementary detection approaches, including dark matter–electron scattering in semiconductors^3^, with recent experimental progress demonstrated in low-threshold electron-recoil searches^4^, as well as phonon-mediated detection in polar materials^5,6^ and magnon-based detection in magnetic systems^7,8^. Together, these developments highlight the growing interplay between particle physics and condensed-matter physics in the search for light dark matter^1^. These distinct channels are not merely technical alternatives, but reflect fundamentally different couplings between dark matter and the internal degrees of freedom of the target material.

From a theoretical perspective, the observable material response is determined not only by the properties of the dark matter particle, such as its mass and velocity distribution, but also by the intrinsic characteristics of the target material. Quantities such as the electronic band structure, dielectric response, phonon spectrum, and magnetic ordering play a central role in shaping the available excitation channels. Effective field theory and linear-response frameworks provide a unified description of these processes, enabling a systematic mapping between dark matter interactions and material-dependent response functions^9,10^. Despite substantial progress in modeling individual detection channels, most existing studies focus on a single material or a single excitation mechanism. As a result, the role of the target material in shaping the leading excitation pathway has not been systematically explored within a unified comparative framework that treats different materials on equal footing. In particular, it remains useful to examine whether realistic materials exhibit genuinely mixed multi-channel responses, or whether their responses are preferentially weighted toward one leading excitation channel under common physical assumptions.

In this work, we address this question by developing a comparative material-dependent framework for sub-GeV dark matter detection. We consider three representative targets—silicon (Si), gallium arsenide (GaAs), and nickel oxide (NiO)—which span semiconducting, polar, and antiferromagnetic material classes. Within a unified multi-channel response model that includes electronic, phononic, and magnonic contributions under identical dark matter assumptions, we systematically compare the response of each material and examine the relative channel preference within the adopted normalized effective-response model. Within this framework, all materials are evaluated under the same dark matter input, ensuring that observed differences arise from the adopted material-response parameterization, effective weights, and common kinematic assumptions rather than from changes in the external kinematic assumptions.

The central objective of this study is not to identify a universally optimal target material or to predict absolute experimental rates, but rather to examine how different material classes may preferentially emphasize distinct excitation pathways within a normalized effective-response model. By decomposing the total response into channel-resolved contributions and introducing a dominance criterion, the analysis provides a transparent way to compare electron-, phonon-, and magnon-sensitive target classes under common kinematic assumptions.

This perspective may be useful for target-selection considerations. Instead of relying on a single target material, a multi-target strategy combining semiconductors, polar materials, and magnetic systems may provide broader coverage of possible sub-GeV dark matter excitation signatures.

The novelty of the present work therefore lies not in introducing a new microscopic excitation channel, but in organizing electronic, phononic, and magnonic responses within a single normalized comparative framework applied consistently to semiconducting, polar, and magnetic target classes. This unified treatment allows the relative channel preference and its sensitivity to effective material weighting to be examined in a transparent and reproducible way.

The remainder of this paper is organized as follows. Section “Physical motivation” introduces the physical motivation. Section “Theoretical framework” presents the theoretical framework. Section “Material-dependent detection channels” discusses the selected material channels. Sections “Scattering and response model” and “Comparative material-dependent response model” describe the scattering model, normalization, numerical implementation, and comparative response framework. Section “Results and discussion” presents the results, sensitivity tests, and literature-parameter benchmark. Section “Model limitations” discusses model limitations, and Section “Conclusion” concludes the paper.

## Physical motivation

The detection of sub-GeV dark matter requires a departure from traditional nuclear-recoil-based strategies toward mechanisms capable of probing much smaller energy depositions. In this regime, the relevant energy scales are typically in the meV–eV range, where condensed-matter systems provide a natural environment for converting dark matter interactions into observable signals^1,2^.

Unlike heavy dark matter candidates, which primarily interact through nuclear recoils, light dark matter is more efficiently probed through its coupling to low-energy degrees of freedom within the target material. These include electronic excitations, lattice vibrations (phonons), and collective spin excitations (magnons), each of which corresponds to a distinct physical process with its own characteristic energy scale and selection rules^3,5,7^.

The existence of multiple excitation channels raises an important conceptual question: how does a given material distribute the deposited energy among the available degrees of freedom? In principle, a sufficiently complex material could support multiple competing channels, leading to a mixed response in which no single excitation mechanism dominates. However, the intrinsic properties of the material–such as its band structure, dielectric response, and magnetic ordering–may strongly favor one channel over the others.

This observation suggests that the role of the target material is not merely quantitative, in the sense of enhancing or suppressing the normalized response, but also qualitative, in shaping the leading excitation channel within the adopted model. In other words, different materials may exhibit responses preferentially weighted toward different excitation channels, even when subject to the same underlying dark matter input.

From a practical standpoint, this distinction is relevant for target selection and comparative detector-material studies. If a material exhibits a clear preference for a specific excitation channel within the adopted model, then it may be considered as a candidate for probing interaction scenarios associated with that channel. Conversely, if different materials favor different channels, then a multi-target strategy may provide broader coverage of possible interaction signatures.

Motivated by these considerations, the present work adopts a comparative approach in which multiple materials are analyzed within a unified framework. By evaluating the response of each target under identical assumptions and decomposing the resulting response into channel-resolved contributions, it becomes possible to identify the leading excitation pathway within the adopted assumptions in a systematic and transparent manner.

This motivates the development of the multi-channel response model introduced in the following sections, where the material-dependent nature of the detection process is made explicit.

## Theoretical framework

The interaction of sub-GeV dark matter with condensed-matter targets is most naturally described in terms of energy deposition into low-energy excitations. In this regime, the relevant observable is not a nuclear recoil, but rather the rate at which dark matter induces transitions among the internal degrees of freedom of the material, such as electronic states, lattice vibrations, or spin excitations.

Within a general framework, the differential event rate per unit target mass can be expressed as

1 $$\begin{aligned} \frac{dR}{d\omega } = \frac{\rho _\chi }{m_\chi } \int d^3 v \, f(\textbf{v}) \, \frac{d\Gamma }{d\omega }(\textbf{v}, \omega ), \end{aligned}$$

where $\rho _\chi$ is the local dark matter density, $m_\chi$ is the dark matter mass, $f(\textbf{v})$ is the velocity distribution in the laboratory frame, and $d\Gamma /d\omega$ represents the differential scattering rate into excitations of energy $\omega$.

The quantity $d\Gamma /d\omega$ encodes the microscopic interaction between dark matter and the target and can be factorized into a model-dependent interaction term and a material-dependent response function. In condensed-matter systems, this response is conveniently described using linear-response theory, where the relevant information is contained in dynamical structure factors or response functions associated with the degrees of freedom of interest^9,10^.

For a given excitation channel *i*, the rate can be schematically written as

2 $$\begin{aligned} R_i \propto \int d\omega \, \mathcal {K}_i(m_\chi , \omega ) \, S_i(\omega ), \end{aligned}$$

where $\mathcal {K}_i$ represents the kinematic and interaction-dependent kernel, and $S_i(\omega )$ is the material-dependent response function corresponding to the excitation channel. The index *i* labels the type of excitation, such as electronic ($i=e$), phononic ($i=\textrm{ph}$), or magnonic ($i=\textrm{mag}$).

This formulation highlights a key feature of sub-GeV dark matter detection in condensed matter: the observable signal is determined by the interplay between dark matter kinematics and the intrinsic excitation structure of the material. While the interaction kernel $\mathcal {K}_i$ is common to all targets under fixed assumptions, the response functions $S_i(\omega )$ vary significantly between materials, reflecting differences in band structure, lattice dynamics, and magnetic ordering.

In the present work, we adopt a simplified but physically motivated approach in which the detailed response functions are replaced by effective channel-dependent contributions that capture the dominant behavior of each excitation mechanism. This allows for a transparent comparison between different materials while retaining the essential physics of multi-channel energy deposition.

The total response of a given material is then constructed as a combination of contributions from the relevant excitation channels, which are evaluated under identical dark matter assumptions. This framework provides the basis for the comparative multi-channel model introduced in the following section.

## Material-dependent detection channels

### Silicon (Si): electronic excitations

Silicon is one of the standard benchmark materials in light dark matter direct-detection studies based on electronic excitations^3^. Its importance arises from the possibility that a sub-GeV dark matter particle may transfer sufficient energy to promote an electron from an occupied state into the conduction band, thereby producing an observable ionization signal. Since the characteristic electronic excitation scale in silicon is much smaller than the recoil energies relevant to conventional nuclear-recoil experiments, silicon provides a natural platform for probing dark matter candidates in the low-mass regime.

Another advantage of silicon is that its electronic structure has been extensively studied in the literature, making it an appropriate reference material for comparative analyses^3^. In the present work, silicon is therefore chosen to represent the electron-excitation channel and to serve as the baseline target for comparison with phonon- and magnon-mediated detection mechanisms.

### Gallium arsenide (GaAs): phonon excitations

Gallium arsenide is selected here as a representative polar material for phonon-mediated dark matter detection^5,6^. In such a target, the deposited dark matter energy can be absorbed by collective lattice vibrations rather than by electronic transitions. Because phonon excitations generally involve lower characteristic energies than electronic excitations, GaAs is particularly suitable for probing lighter dark matter candidates whose available kinetic energy is limited.

The relevance of GaAs also follows from its well-defined lattice dynamics and the presence of phonon modes that can efficiently couple to external perturbations^5,6^. For this reason, it serves in the present comparative framework as the representative material for the phonon-excitation channel, illustrating how lattice degrees of freedom may extend the reach of direct-detection searches toward lower dark matter masses.

### Nickel oxide (NiO): magnon excitations

Nickel oxide is considered in this work as a representative magnetic material for magnon-based dark matter detection^7,8^. In magnetically ordered systems, the deposited energy may excite collective spin-wave modes instead of charge carriers or lattice vibrations. This opens a complementary detection pathway that is particularly relevant when the effective dark matter interaction contains spin-dependent terms.

The antiferromagnetic character of NiO makes it a natural candidate for studying this type of response^8^. Its excitation spectrum is governed by spin ordering and magnetic dynamics, which distinguishes it qualitatively from the electronic response of silicon and the phonon response of GaAs. Accordingly, NiO is used here to represent the magnon-excitation channel and to extend the comparative analysis to spin-sensitive detection prospects.

## Scattering and response model

In order to evaluate the material-dependent detection behavior, we adopt a simplified but physically motivated model for the dark matter scattering rate in each target. The purpose of this model is not to reproduce detailed ab initio calculations, but rather to capture the dominant features of the relevant excitation channels in a transparent and comparable form. In the present formulation, the dark-matter interaction is represented through a common effective kinematic kernel applied consistently to all target materials. The aim is not to specify a complete particle-physics model with a fixed mediator, cross-section normalization, or detector exposure, but to isolate how the adopted material-response parameterization affects the relative channel ranking. The kernel therefore plays the role of a normalized comparative driver, while the material dependence enters through excitation thresholds, effective response kernels, and channel weights. The response rates reported below are therefore normalized comparative responses in arbitrary units. They should not be interpreted as exposure-normalized event rates or as experimental sensitivity projections. No detector efficiency, exposure, background model, or exclusion-limit construction is included. This clarification is important because the present study is intended as a channel-ranking and material-comparison exercise rather than a replacement for microscopic calculations of electronic, phononic, or magnonic structure factors.

For each material, the total response is constructed from contributions associated with the primary excitation mechanisms identified in Section Material-Dependent Detection Channels. These correspond to electronic excitations in silicon, phonon excitations in gallium arsenide, and magnon excitations in nickel oxide. Under fixed dark matter kinematics, the relative importance of these channels is determined by the adopted material-response parameterization, excitation thresholds, and effective channel weights rather than by variations in the interaction model.

### Silicon: electronic response

For silicon, the dominant contribution is assumed to arise from electronic excitations, consistent with previous studies of dark matter–electron scattering in semiconductors^3^. The response is modeled using an effective electronic rate that depends on the available phase space for electron transitions across the band gap. This approach captures the leading behavior of the electronic channel without requiring detailed band-structure calculations.

### Gallium arsenide: phonon response

For gallium arsenide, we consider a phonon-mediated response in which the deposited energy excites lattice vibrations. Such processes have been shown to provide sensitivity to low-energy dark matter interactions in polar materials^5,6^. The phonon contribution is modeled through an effective rate that reflects the characteristic energy scale of lattice excitations and their coupling to external perturbations.

### Nickel oxide: magnon response

For nickel oxide, the dominant channel is taken to be magnon excitation, corresponding to collective spin-wave modes in the antiferromagnetic lattice. Magnon-based detection has recently been proposed as a promising avenue for probing light dark matter interactions with spin degrees of freedom^7,8^. The magnon response is therefore represented by an effective rate that captures the relevant spin excitation spectrum.

### Total response construction

The total response for each material is obtained by combining the contributions from the relevant excitation channels under a unified set of dark matter assumptions. In the present model, all materials are evaluated using the same kinematic inputs, ensuring that any differences in the resulting rates originate from the structure of the material response itself.

This simplified treatment provides a consistent basis for comparing different materials within a common framework, while retaining the essential physical distinctions between electronic, phononic, and magnonic detection mechanisms. The resulting responses are then used in Section Comparative Material-Dependent Response Model to construct the material-dependent weighting model and to analyze the relative dominance of each excitation channel.

## Comparative material-dependent response model

This section presents the comparative framework used to analyze the responses of silicon (Si), gallium arsenide (GaAs), and nickel oxide (NiO) in the sub-GeV dark matter regime. The aim is not merely to compare total event rates, but to determine how the intrinsic properties of each material shape the dominant low-energy excitation mechanism. In particular, the model is designed to identify whether the observable response is governed primarily by electronic, phononic, or magnonic excitations under a common set of dark matter assumptions.

### Common dark matter assumptions

To place all target materials on equal footing, we adopt the same dark matter input throughout the analysis. The local dark matter density is denoted by $\rho _\chi$, the dark matter mass by $m_\chi$, and the velocity distribution by *f*(*v*), normalized according to

3 $$\begin{aligned} \int d^3v\, f(v) = 1. \end{aligned}$$

The analysis is performed over a common sub-GeV mass interval,

4 $$\begin{aligned} m_\chi \in [m_{\chi ,\min },\, m_{\chi ,\max }], \end{aligned}$$

with identical halo assumptions applied to all materials. In the numerical implementation we use $m_\chi \in [10^{-3},1]~\textrm{GeV}$, $\rho _\chi =0.3~\textrm{GeV}/\textrm{cm}^3$, and standard halo-speed inputs $v_0=220~\mathrm {km/s}$, $v_{\textrm{esc}}=544~\mathrm {km/s}$, and $v_e=232~\mathrm {km/s}$. Under this setup, differences in the normalized response arise from the material response parameterization and channel weights rather than from changes in the external halo assumptions.

### Channel-resolved response structure

For each material $X$, where

5 $$\begin{aligned} X \in \{\textrm{Si}, \textrm{GaAs}, \textrm{NiO}\}, \end{aligned}$$

the total response is decomposed into three contributions,

6 $$\begin{aligned} R_{\textrm{tot}}^{(X)}(m_\chi ) = R_e^{(X)}(m_\chi ) + R_{\textrm{ph}}^{(X)}(m_\chi ) + R_{\textrm{mag}}^{(X)}(m_\chi ), \end{aligned}$$

where $R_e^{(X)}$, $R_{\textrm{ph}}^{(X)}$, and $R_{\textrm{mag}}^{(X)}$ represent the electronic, phononic, and magnonic responses, respectively.

This decomposition allows the same material to be described within a unified multi-channel framework, even if one channel is expected to dominate physically. It also provides a transparent basis for comparing materials that naturally favor different types of low-energy excitations.

### Material-dependent weighting of excitation channels

Although all three channels are formally retained in the model, their relative importance is not expected to be the same for all materials. To account for this in a transparent phenomenological way, we introduce literature-guided effective material weights and write

7 $$\begin{aligned} R_e^{(X)}(m_\chi ) = w_e^{(X)}\,\widetilde{R}_e^{(X)}(m_\chi ), \end{aligned}$$

8 $$\begin{aligned} R_{\textrm{ph}}^{(X)}(m_\chi ) = w_{\textrm{ph}}^{(X)}\,\widetilde{R}_{\textrm{ph}}^{(X)}(m_\chi ), \end{aligned}$$

9 $$\begin{aligned} R_{\textrm{mag}}^{(X)}(m_\chi ) = w_{\textrm{mag}}^{(X)}\,\widetilde{R}_{\textrm{mag}}^{(X)}(m_\chi ), \end{aligned}$$

where $\widetilde{R}_i^{(X)}$ denotes the unweighted response associated with the corresponding interaction kernel and material response function, while $w_i^{(X)}$ represents the effective relative weight assigned to channel $i$ for material $X$. These weights are not universal material constants. They summarize, at the phenomenological level, the expectation that Si is primarily used as an electronic-excitation semiconductor benchmark, GaAs as a polar phonon-sensitive target, and NiO as an antiferromagnetic magnon-sensitive target. Representative semiconductor and material properties used in the present analysis are consistent with standard solid-state references^11^.

The adopted material parameters used in the present model are summarized in Table 1, while the effective channel weights are listed in Table 2. Because these weights influence the channel ranking, their impact is explicitly tested in the sensitivity analysis below.

**Table 1:** Material parameters used in the comparative model.

Material	Parameter	Value
Si	Band gap $E_g$	$1.1242~\textrm{eV}$
Si	Relative permittivity $\varepsilon _r$	$11.7$
GaAs	Band gap $E_g$	$1.42~\textrm{eV}$
GaAs	LO phonon energy $\omega _{\textrm{LO}}$	$0.0365~\textrm{eV}$
GaAs	High-frequency dielectric constant $\varepsilon _\infty$	$10.9$
GaAs	Static dielectric constant $\varepsilon _0$	$12.9$
NiO	Effective gap $E_g$	$3.6~\textrm{eV}$
NiO	Magnon energy $\omega _{\textrm{mag}}$	$0.00443~\textrm{eV}$
NiO	Effective phonon scale $\omega _{\textrm{ph}}$	$0.050~\textrm{eV}$

**Table 2:** Literature-guided effective channel weights used in the normalized response model. These values are phenomenological weights, not universal material constants.

Material	$w_e$	$w_{\textrm{ph}}$	$w_{\textrm{mag}}$
Si	$1.00$	$0.08$	$0.005$
GaAs	$0.015$	$2.50$	$0.003$
NiO	$0.002$	$0.06$	$3.00$

### Weight-sensitivity protocol

Since the effective channel weights enter directly into the channel-resolved rates, we test the stability of the dominant-channel assignment by perturbing the baseline weights. For each material, the baseline dominant-channel weight is reduced while the two competing channel weights are increased. Two adverse perturbations are considered:

10 $$\begin{aligned} w_\textrm{dom}\rightarrow 0.75w_\textrm{dom},\qquad w_\textrm{sub}\rightarrow 1.25w_\textrm{sub}, \end{aligned}$$

and

11 $$\begin{aligned} w_\textrm{dom}\rightarrow 0.50w_\textrm{dom},\qquad w_\textrm{sub}\rightarrow 1.50w_\textrm{sub}. \end{aligned}$$

We also include a unit-weight test, $w_e=w_\textrm{ph}=w_\textrm{mag}=1$, to separate the role of the material-guided weights from the role of the unweighted response kernels. This test is not presented as a physically calibrated model, but as a diagnostic for weight dependence.

### Numerical implementation and reproducibility

The numerical implementation follows the normalized response model described above. The dark matter mass is sampled over the interval

$$m_\chi \in [10^{-3},1]~\textrm{GeV}$$

using 200 logarithmically spaced points. The momentum variable is evaluated on a uniform grid

$$q \in [10^{-4},1],$$

with 600 grid points. For each mass value and channel, the corresponding normalized response integral is evaluated using the trapezoidal rule over the $q$-grid. The same mass grid, momentum grid, halo inputs, effective kernels, material parameters, and channel weights are used for all materials to ensure that the reported differences arise only from the adopted material-response parameterization.

The numerical setup used in the calculations is summarized in Table 3.

**Table 3:** Numerical setup used for the normalized comparative calculations.

Quantity	Value/Description
Dark matter mass range	$10^{-3}$–$1$ GeV
Number of mass points	200 logarithmically spaced points
Momentum grid	$q \in [10^{-4},1]$
Number of $q$-grid points	600
Integration method	Trapezoidal rule
Local dark matter density	$0.3~\mathrm {GeV/cm^3}$
Halo-speed inputs	$v_0=220$ km/s, $v_\textrm{esc}=544$ km/s, $v_e=232$ km/s
Output normalization	Normalized response rates in arbitrary units

### Effective response kernels versus microscopic response functions

The present model does not compute microscopic response functions from first-principles electronic, phononic, or magnonic structure. Accordingly, the response quantities used here are treated as effective surrogate kernels rather than microscopic material response functions. This distinction is summarized in Table 4.

**Table 4:** Relation between microscopic response quantities and the effective surrogate kernels used in the present phenomenological model.

Channel	Microscopic quantity not computed	Effective surrogate used here
Electronic	Band-structure transition matrix elements	Gap-controlled electronic kernel
Phononic	Phonon dispersion and dynamical matrix	LO-phonon/effective threshold kernel
Magnonic	Spin-wave spectrum and spin structure factor	Magnon-energy effective kernel

Thus, the present calculation should not be interpreted as a replacement for ab initio response-function calculations. Its role is to provide a normalized and reproducible phenomenological comparison of channel preferences under common assumptions.

### Threshold-energy constraints

A central ingredient in the comparison is the characteristic excitation threshold of each channel. Let

12 $$\begin{aligned} \omega _{\textrm{th},e}^{(X)},\qquad \omega _{\textrm{th},\textrm{ph}}^{(X)},\qquad \omega _{\textrm{th},\textrm{mag}}^{(X)} \end{aligned}$$

denote the effective thresholds for the electronic, phononic, and magnonic channels in material $X$.

For a given dark matter mass $m_\chi$, a channel contributes only if the deposited energy satisfies

13 $$\begin{aligned} \omega \ge \omega _{\textrm{th},i}^{(X)}, \qquad i \in \{e,\textrm{ph},\textrm{mag}\}. \end{aligned}$$

Using the kinematic condition

14 $$\begin{aligned} \omega = q \cdot v - \frac{q^2}{2m_\chi }, \end{aligned}$$

one sees that the accessibility of a specific channel depends jointly on the dark matter kinematics and the excitation scale of the target material.

This threshold dependence is especially important in the sub-GeV regime, where the available kinetic energy is small and low-energy excitations become the relevant observables.

### Fractional channel contributions

To quantify the relative importance of each excitation mechanism, we define the normalized channel fractions

15 $$\begin{aligned} \eta _i^{(X)}(m_\chi )=\frac{R_i^{(X)}(m_\chi )}{R_{\textrm{tot}}^{(X)}(m_\chi )}, \qquad i \in \{e,\textrm{ph},\textrm{mag}\}. \end{aligned}$$

These fractions satisfy

16 $$\begin{aligned} \eta _e^{(X)}(m_\chi )+\eta _{\textrm{ph}}^{(X)}(m_\chi )+\eta _{\textrm{mag}}^{(X)}(m_\chi )=1. \end{aligned}$$

The quantities $\eta _i^{(X)}$ provide a direct measure of how strongly a given material favors one channel over the others. They are particularly useful for determining whether a target exhibits a genuinely mixed multi-channel response or an effectively single-channel-dominated response within the adopted phenomenological framework.

### Dominant excitation indicator

To identify the leading excitation mechanism in each material, we define the dominant excitation indicator

17 $$\begin{aligned} D^{(X)}(m_\chi )=\arg \max \left\{ R_e^{(X)}(m_\chi ),\,R_{\textrm{ph}}^{(X)}(m_\chi ),\,R_{\textrm{mag}}^{(X)}(m_\chi )\right\} . \end{aligned}$$

This quantity determines which channel gives the largest contribution to the total response at fixed dark matter mass.

In the present work, the role of this indicator is primarily classificatory. Rather than emphasizing internal switching between channels within a single material, the model is designed to determine which excitation mechanism is intrinsically preferred by each target over the considered mass interval.

### Relative normalized material response

In addition to the internal decomposition of each material, it is useful to compare the relative normalized response of the three targets within the adopted model. For this purpose, we define the relative normalized material response measure

18 $$\begin{aligned} S_X(m_\chi )=\frac{R_{\textrm{tot}}^{(X)}(m_\chi )}{\max \limits _{Y\in \{\textrm{Si},\textrm{GaAs},\textrm{NiO}\}}R_{\textrm{tot}}^{(Y)}(m_\chi )}, \end{aligned}$$

so that

19 $$\begin{aligned} 0 \le S_X(m_\chi ) \le 1. \end{aligned}$$

This quantity indicates which material gives the largest normalized total response at a given dark matter mass under identical external assumptions within the adopted model.

### Interpretation of the comparative framework

The comparative framework developed here combines three complementary layers:

1. a common dark matter input shared by all materials,
2. a channel-resolved decomposition of the total response in each target,
3. a material-dependent dominance criterion that identifies the preferred excitation channel.

From this perspective, the purpose of the model is not simply to rank normalized responses, but to examine how different condensed-matter systems may favor different low-energy excitation channels within the adopted phenomenological framework. The numerical results in the next section are therefore interpreted in terms of material-dependent channel preferences within the adopted model rather than as a model-independent performance ranking of the materials.

## Results and discussion

In this section, we present the numerical results obtained from the comparative multi-channel response model introduced in Section Comparative Material-Dependent Response Model. The analysis focuses on both the total response and the internal channel decomposition for silicon (Si), gallium arsenide (GaAs), and nickel oxide (NiO) across the sub-GeV dark matter mass range.

The results are interpreted as normalized comparative trends within the adopted phenomenological model. Particular attention is given to whether the channel ranking remains stable when the effective material weights are perturbed.

### Total response comparison

**Fig. 1:**
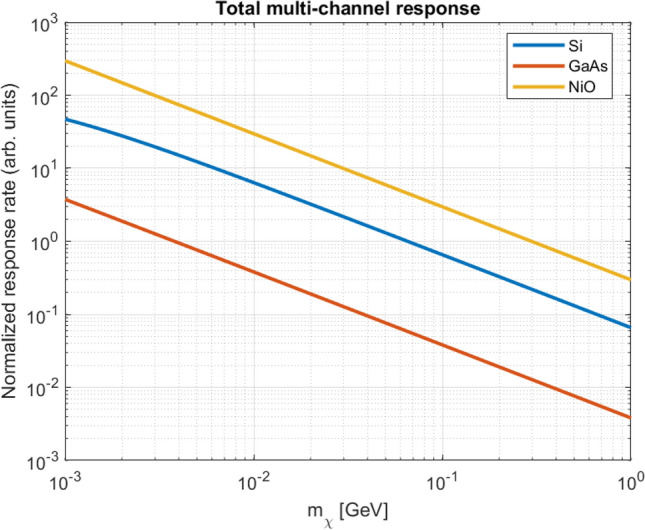
Normalized total multi-channel response for Si, GaAs, and NiO as a function of the dark matter mass. The vertical axis is in arbitrary units and is used for relative comparison only.

Figure 1 shows the normalized total multi-channel response for the three materials as a function of the dark matter mass. Within the baseline parameterization, NiO gives the largest normalized response, followed by Si and then GaAs.

The absence of curve crossings in the baseline calculation indicates that this ordering is stable within the specified parameter set. It should not, however, be interpreted as an absolute experimental rate hierarchy, since the calculation does not include detector exposure, efficiency, backgrounds, or microscopic ab initio response functions.

### Channel decomposition in silicon

**Fig. 2:**
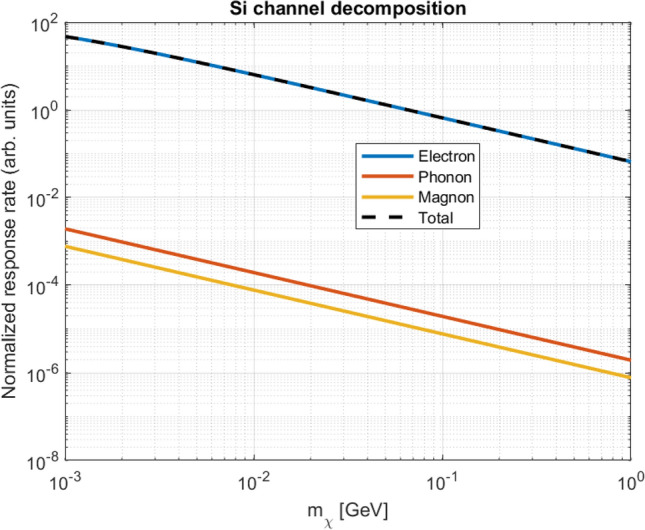
Channel decomposition of the silicon response. The normalized total response closely follows the electronic contribution within the baseline parameterization, while phonon and magnon channels remain strongly suppressed.

The channel-resolved response of silicon is shown in Figure 2. The total response is seen to follow the electronic contribution closely within the baseline parameterization, while the phonon and magnon channels remain suppressed by several orders of magnitude across the full mass range.

This result indicates that silicon exhibits an effectively single-channel-dominated response within the adopted normalized model, with the electronic contribution providing the leading channel over the considered mass range. The dominance of the electronic channel is consistent with the semiconductor nature of silicon and with the role of the band-gap-controlled excitation structure in determining its response.

### Channel decomposition in GaAs

**Fig. 3:**
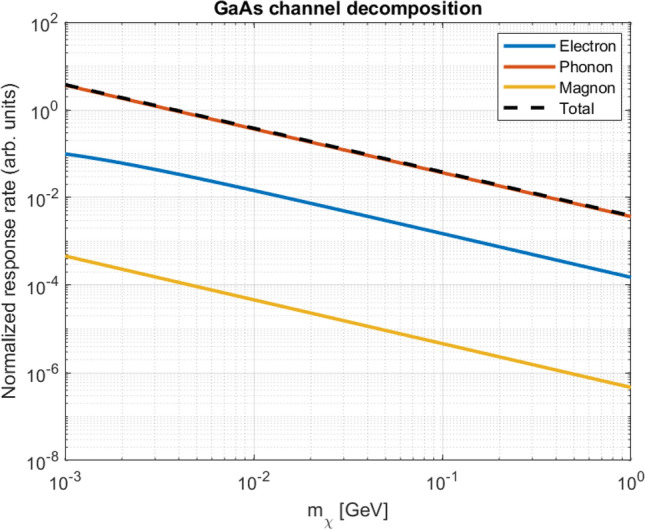
Channel decomposition of the GaAs response. The phonon channel gives the leading normalized contribution across the scanned interval under the baseline parameterization, while the electronic and magnonic channels remain subleading.

Figure 3 displays the channel decomposition for GaAs. In contrast to silicon, the total response in GaAs is dominated by the phonon channel, with the phonon contribution nearly coinciding with the total rate. The electronic channel remains subleading, and the magnon channel is negligible.

This behavior reflects the polar nature of GaAs and the important role of longitudinal optical phonons in mediating low-energy response. The results therefore suggest that GaAs exhibits a phonon-favored response within the baseline effective-weight parameterization, rather than a strongly mixed multi-channel response.

### Channel decomposition in NiO

**Fig. 4:**
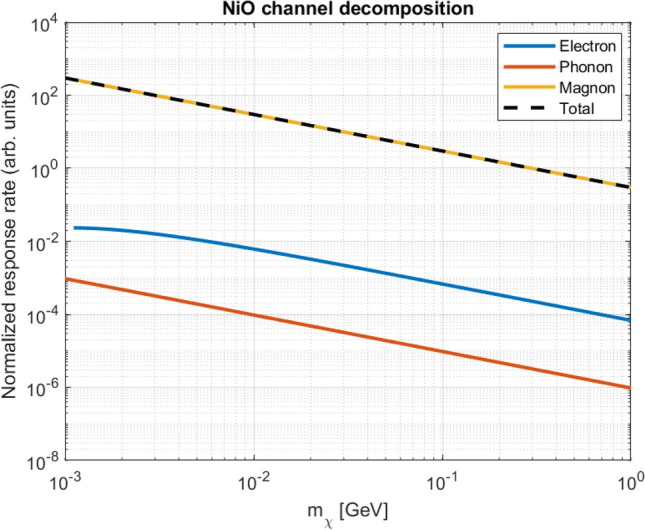
Channel decomposition of the NiO response. The normalized total response is mainly controlled by the magnon contribution within the baseline parameterization, while the electronic and phononic channels remain strongly suppressed.

The response decomposition for NiO is presented in Figure 4. The results indicate a baseline preference for the magnon channel, with the total response mainly controlled by magnon excitations within the adopted model. Both electronic and phononic contributions are suppressed by several orders of magnitude.

This behavior is characteristic of antiferromagnetic materials, where low-energy spin excitations provide an efficient and natural interaction channel. Within the present model, NiO exhibits a magnon-favored response within the adopted phenomenological parameterization.

### Fractional channel contributions

**Fig. 5:**
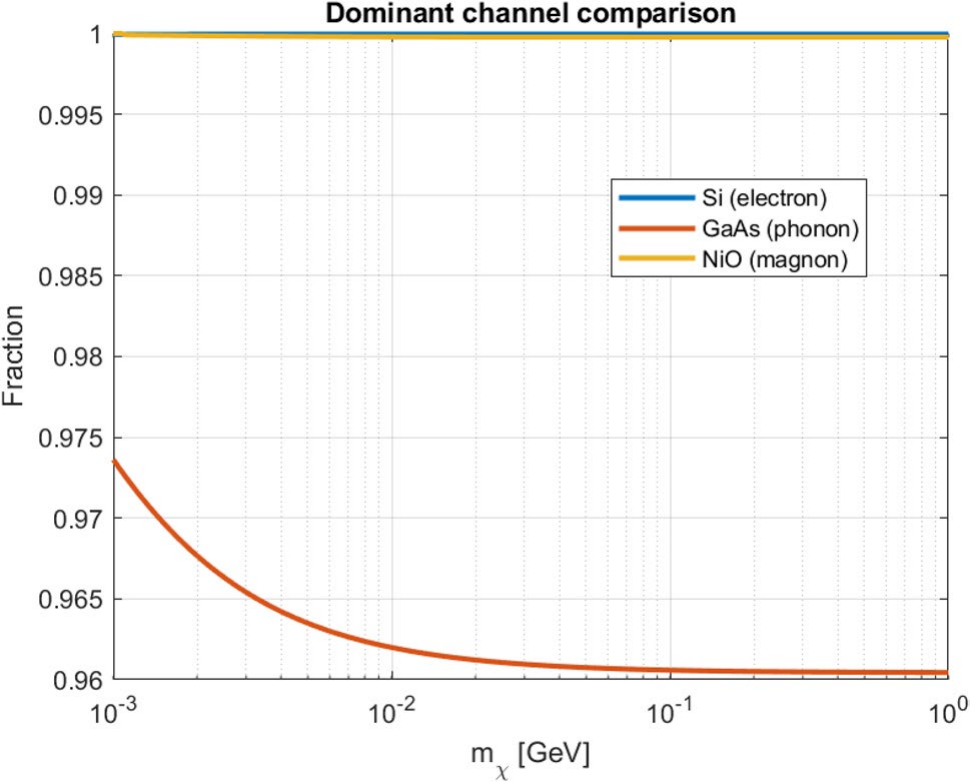
Baseline fractional contributions of the leading excitation channels in Si, GaAs, and NiO. These fractions illustrate the channel ranking within the adopted phenomenological effective-weight model and should not be interpreted as model-independent material response functions.

The dominance pattern becomes even clearer in Figure 5, which shows the fractional contributions of the leading channels in the three materials. The results demonstrate that the baseline fractions indicate strong electronic dominance in Si, phonon dominance in GaAs, and magnon dominance in NiO throughout the considered mass range.

This figure indicates, within the baseline parameterization, that the three materials do not behave as strongly mixed multi-channel systems. Instead, each material exhibits an effectively single-channel-dominated response within the adopted model, with one excitation mechanism providing the leading contribution in the baseline normalized response. This conclusion is also directly consistent with the numerical values in the results table, where the dominant fractions remain close to unity for the leading channel in each material.

### Dominant excitation channels and physical implications

The dominant excitation channel for each material is summarized in Table 5. The numerical results show a clear baseline pattern: silicon favors electronic excitations, GaAs favors phonon excitations, and NiO favors magnon excitations across the scanned mass interval under the baseline parameterization.

**Table 5:** Dominant excitation channel for each material across the sub-GeV mass range.

Material	Dominant Channel
Si	Electron
GaAs	Phonon
NiO	Magnon

Under the baseline effective-weight parameterization, each material response is approximately single-channel dominated within the considered mass range. Silicon is dominated by electronic excitations, GaAs by phonon excitations, and NiO by magnon excitations.

This behavior should be interpreted conditionally: it reflects the combined effect of the adopted response kernels, excitation thresholds, and effective material weights. The following sensitivity test therefore examines whether the same classification survives adverse perturbations of the weights.

### Weight-sensitivity results

Table 6 summarizes the stability of the baseline dominant-channel assignment under adverse perturbations of the effective weights. The Si and NiO classifications remain unchanged even under the adverse $\pm 50\%$ perturbation. The GaAs phonon classification also remains stable under this adverse perturbation, although the leading phonon fraction is reduced. In the diagnostic unit-weight case, however, GaAs switches away from the phonon-dominated classification. This confirms that the GaAs result depends on retaining material-guided channel weighting and should not be presented as a model-independent consequence of kinematics alone.

**Table 6:** Weight-sensitivity summary. The percentage indicates the fraction of sampled dark matter masses for which the baseline dominant channel remains dominant.

Material	Scenario	Dominant channel	Baseline dominance retained
Si	Baseline	Electron	100%
Si	Adverse $\pm 25\%$	Electron	100%
Si	Adverse $\pm 50\%$	Electron	100%
Si	Unit weights	Electron	100%
GaAs	Baseline	Phonon	100%
GaAs	Adverse $\pm 25\%$	Phonon	100%
GaAs	Adverse $\pm 50\%$	Phonon	100%
GaAs	Unit weights	Electron	0%
NiO	Baseline	Magnon	100%
NiO	Adverse $\pm 25\%$	Magnon	100%
NiO	Adverse $\pm 50\%$	Magnon	100%
NiO	Unit weights	Magnon	100%

### Literature-parameter channel-preference benchmark

To further check that the normalized model is not merely producing arbitrary channel assignments, we performed a qualitative benchmark using literature-guided material parameters and channel expectations. This test is not a direct rate-to-rate validation, because the present responses are normalized and reported in arbitrary units. Instead, it checks whether the model reproduces the expected leading excitation class for each representative material.

**Table 7:** Literature-parameter channel-preference benchmark. The comparison is qualitative and tests channel preference rather than absolute event rates.

Material	Literature-guided expectation	Model leading channel	Mean leading fraction
Si	Electron-sensitive semiconductor target^3^	Electron	0.99996
GaAs	Polar phonon-sensitive target^5,6^	Phonon	0.96255
NiO	Magnetic/magnon-sensitive target^7,8^	Magnon	0.99980

The benchmark shows qualitative consistency between the present normalized framework and the expected leading excitation trends of the selected material classes. It should be interpreted only as a qualitative channel-preference check, not as experimental validation or as a direct comparison with absolute event-rate calculations. A direct numerical comparison with previous rate calculations would require the same interaction model, cross-section normalization, microscopic response functions, detector thresholds, and exposure assumptions.

From a practical perspective, these findings support the idea that no single material class should be treated as universally representative of all low-energy dark matter interaction channels within the adopted phenomenological model. Instead, semiconductors, polar materials, and magnetic systems should be viewed as complementary components of a broader detection strategy. In this sense, the present results support a multi-target perspective in which different materials are considered not only in terms of their normalized response strength, but also in terms of their channel-preference information in the sub-GeV regime.

## Model limitations

The present model has several limitations that delimit the interpretation of the results. First, the response functions are not computed from microscopic electronic band structures, phonon dispersions, or magnon spectra. Instead, the calculation uses effective surrogate kernels designed for normalized channel comparison. Second, the plotted rates are normalized comparative responses in arbitrary units, not absolute event rates per exposure. Third, the effective channel weights are phenomenological and literature-guided; they are not universal material constants and depend on the assumed interaction scenario. Fourth, the literature-parameter benchmark reported above is a qualitative channel-preference check rather than a direct numerical validation against absolute rates. Fifth, the model does not include detector efficiencies, backgrounds, experimental thresholds beyond the simplified excitation-scale conditions, or exclusion-limit construction.

Finally, disorder effects such as vacancies, grain boundaries, impurities, and finite-temperature broadening are not explicitly included. In practical Si, GaAs, and NiO samples, such defects may change the relative strength of electronic, phononic, or magnonic signatures by introducing localized states, modifying phonon or magnon linewidths, altering coupling strengths, or shifting effective excitation thresholds. Therefore, the present results should be interpreted as an ideal-crystalline baseline rather than as a defect-resolved detector prediction. The model should be refined in future work using microscopic response functions and experimentally normalized calculations.

## Conclusion

We have presented a phenomenological comparative framework for examining channel-resolved excitation trends in sub-GeV dark matter detection using Si, GaAs, and NiO as representative semiconducting, polar, and antiferromagnetic targets. The model combines electronic, phononic, and magnonic contributions under common halo and mass-scan assumptions and expresses the results as normalized comparative responses rather than absolute event-rate predictions.

The contribution of the work is therefore methodological and comparative: it provides a common normalized framework for contrasting three material classes and testing how stable the inferred channel preferences are under controlled perturbations of the effective weights.

Within the baseline literature-guided effective-weight parameterization, the baseline model classifies Si as electron-dominated, GaAs as phonon-dominated, and NiO as magnon-dominated over the scanned mass interval. A new weight-sensitivity analysis shows that these assignments remain stable in the tested adverse $\pm 25\%$ and $\pm 50\%$ perturbation scenarios of the effective weights. At the same time, the unit-weight diagnostic demonstrates that the GaAs classification is conditional on including material-guided channel weighting. This result clarifies that the proposed classification is not a model-independent proof of universal material dominance, but a controlled comparison within the stated phenomenological assumptions.

In addition, the literature-parameter channel-preference benchmark provides only a qualitative consistency check, indicating that the adopted normalized framework is consistent with the expected leading excitation trends of the selected representative materials, namely electronic response in Si, phononic response in GaAs, and magnonic response in NiO. This benchmark is not a rate-level validation, but it strengthens the interpretation of the model as a channel-preference comparison.

The main implication is therefore cautious but useful: different classes of condensed-matter targets can preferentially emphasize different low-energy excitation signatures, and a multi-target strategy remains a natural way to broaden coverage of possible sub-GeV dark matter interaction signatures. Future work should replace the effective response kernels with microscopic or ab initio electronic, phononic, and magnonic response functions, include detector-level normalization, and compare directly with experimental sensitivity projections and exclusion limits.

## Data Availability

No new experimental or observational data were generated in this study. The numerical results supporting the findings of this work are available from the author upon reasonable request.
